# Book Notices

**Published:** 1894-09

**Authors:** 


### Book Notices.

THE MODERN CLIMATE TREATMENT OF INVALIDS WITH PULMONARY CONSUMPION IN SOUTHERN CALIFORNIA. By P. C Remondino, M. D., Member of the American Medical Associa tion, American Public Health Association, ex-Vice-President California State Medical Society, etc., etc., etc. 126 pages. Illustrated. Price, paper 25c., cloth 50c. George S. Davis, Publisher, Detroit, Mich.

This book is written in Dr. Remondino's peculiarly interesting style, and is a short and clear statement of the especial advantages that Southern Califonia offers to the invalid, and particularly to those suffering from chronic lung troubles. The effect of this glorious climate on diseases other than of the lungs is clearly stated, and the author calls especial attention to the errors that have obtained very largely to the effect that those suffering from diseases of the kidneys are injured by a residence in California. A flat denial of these erroneous reports is entered, and Dr. Remondino brings forward proofs of the fact that the climate furnishing the greatest equability, with a rather high relative humidity, and a moderately low temperature, is the one most favorable for the prevention of diseases of the kidneys. One chapter is devoted to the "Cost of living, and other details," and in it some valuable information is furnished to those who contemplate going to California in search of health. To such we commend this little volume most cordially. H.

---

A TEXT-BOOK ON DISEASES OF THE EYE. By Henry D. Noyes, A. M., M. D., Professor of Ophthalmology and Otology in Bellevue Hospital Medical College; Executive Surgeon to the New York Eye and Ear Infirmary; recently President of the American Ophthalmological Society; member of the New York Ophthalmological Society; recently Vice-President of the New York Academy of Medicine; permanent member of the New York State Medical Society; member of the American Medical Association, etc., etc. Second and Revised Edition Illustrated by 5 Chromo-lithographic Plates; 10 Plates in black and colors, and 269 Wood Engravings. Price, cloth, $6.00; sheep, $7.00. New York: Wm. Wood & Co. 1894.

It is unnecessary to commend a work so widely and favorably known. The second edition, making its appearance so soon after the first, is sufficient evidence of its worth. The author's many years of experience as a teacher of ophthalmology in one of our largest medical schools, has taught him to know just what the student and practitioner want in a text-book of that science. He has most judiciously drawn the line between the facts and doctrines necessary to be presented and unnecessary details that would add inconveniently to the bulk of such a book. This work has no superior among the many new text-books on diseases of the eye, which have of late appeared.

The illustrations and plates are numerous and well adapted to their purpose. H. L. H.

A PRACTICAL TREATISE ON THE DISEASES OF THE HAIR AND SCALP. By George Thomas Jackson, M. D., Professor of Dermatology, Woman's Medical College, N. Y. Infirmary; Chief of Clinic and Instructor in Dermatology, College of Physicians and Surgeons; Consulting Dermatologist, Presbyterian Hospital, etc., etc., etc. New, revised and enlarged edition. 414 pages. Illustrated. Price, cloth $2.75. E. B. Treat publisher, 5 Cooper Union, New York City.

In 1887 the first edition of this work appeared, and it met with so favorable a reception at the hands of the profession, that a second edition has become necessary, and the author expresses the hope that it will meet with as much favor from medical men as the first edition.

In this edition every page of the old edition has been revised and corrected; new articles upon folliculitis decalvans, lepothrix, and aplasia pilorum propria, and many new sections to the old chapters have been added. The bibliography has been carefully revised, enlarged and brought down to date; a number of new illustrations have been inserted in the text. The book is a valuable one, and with the many improvements that have been made in this second edition it should be more popular with the profession than its popular predecessor. H.

---

SYLLABUS OF THE OBSTETRICAL LECTURES IN THE MEDICAL DEPARTMENT OF THE UNIVERSITY OF PENNSYLVANIA. By Richard C. Norris, A. M., M. D., Demonstrator of Obstetrics, University of Pennsylvania; Assistant Obstetrician, University Maternity; Physician to the Methodist Episcopal Hospital; Obstetrical Registrar, Philadelphia Hospital, etc. Third edition. 222 pages. Price, cloth $2.00. W. B. Saunders, publisher, 925 Walnut St., Philadelphia, 1894.

This work was first prepared to meet the wants of the students in the Medical Department of the University of Pennsylvania, but it has by its merits grown beyond this circumscribed field, and has come into general use among medical students of other schools. It has now reached its third edition, and with the important additions that have been made it will doubtless grow more rapidly into public favor. H.

---

MR. SAUNDERS is pleased to announce, as in active preparation, his *New Aid Series of Manuals for Students and Practitioners.* As publisher of the *Standard Series of Question Compends*, together with an intimate relation with leading members of the medical profession, Mr. Saunders has been enabled to study, progressively, the essential desideratum in practical "self-helps" for students and physicians.

This study has manifested that, while the published "Question Compends" earn the highest appreciation of students, whom they serve in reviewing their studies preparatory to examination, there is special need of thoroughly reliable hand-books on the leading branches of medicine and surgery, each subject being compactly and authoritatively written, and exhaustive in detail, without the introduction of cases and foreign subject matter which so largely expand ordinary text-books.

The *Saunders' Aid Series* will not merely be condensations from present literature, but will be ably written by well-known authors and practitioners, most ot them being teachers in representative American colleges. This new series, therefore, will form an admirable collection of advanced lectures, which will be invaluable aids to students in reading and in comprehending the contents of "recommended" works.

Each manual comprising about 250 pages (5½x8 inches), will further be distinguished by the beauty of the new type; by the quality of the paper and printing; by the copious use of illustrations; by the attractive binding in cloth; and by the extremely low price, which will uniformly be $1.25 per volume.

---

RECOLLECTIONS OF A VIRGINIAN IN THREE WARS—By General Dabney H. Maury. Cloth, $1.50. Scribners, New York. Agent for Texas, Mrs. R. M. Pollard, Houston, Tex., to whom orders may be sent.

We have read this fresh, chatty little volume with much pleasure and satisfaction. It is written in a style to please—free from any attempt at display. It is General Maury's "recollections" told in an easy, conversational manner, just as they came to him, recollections of a great variety of incidents connected with his life, education, and long military service under most varied circumstances. It is not a tale of the war, but "recollections" of camp and field by an active participant. The General seems to have been very fond of hunting all his life, and there is not a recollection but what is embellished with an account of his exploits with the gun, either bird-shooting, duck-shooting or hunting large game, even up to the Mexican lion and the grizzly. To one fond of camp life, as well as to the lover of history, the book can not fail to be most interesting. There is a good deal of inside history in it, too, the version of impartial military officers by an eye witness, or at least a contemporary and participant in the stirring times of the civil war, which have never been published. It is easy to see from General Maury's account that mistakes were made, mistakes in critical times, which had a most important bearing on subsequent events and shaped the end. For instance, when the Yankee fleet run the batteries at Vicksburg, and Grant was about to land at Port Gibson, General Stevenson ordered his division to go out and meet him. This Pemberton countermanded and sent a handful of men (Loring's Brigade), who only annoyed him. "Stevenson," says Gen. Maury, "would have pushed him and his army into the Mississippi river." and especially interesting are the General's reminiscences of our great men, notably, his conversations with General Joseph E. Johnston, to whom he seems to have been much devoted. The General thinks, as most of the soldiers thought, that it was a great mistake to remove Johnston. It seems to us evident that General Maury was not a great admirer of President Davis, even from away back, for of all the West Pointers he has something to say except Jeff Davis; he hardly mentions him either at West Point, in Mexico, or later, as president. To the survivors of Hood's army, especially to the soldiers of Ross' and Ector's brigades, the little book will be most interesting. It is just like talking it over with the General, not only the fighting, but the camp life, with its incidents, is brought vividly before the reader and interspersed with a good deal of "life in the wild west." Send your $1.50 to *his daughter*, Mrs. Pollard, and get the book. It will please you.

---

A TEXT-BOOK OF THE DISEASES OF WOMEN—By Henry J. Garrigues, A. M., M. D., Professor of Obstetrics in the New York Post-Graduate Medical School and Hospital; Gynecologist to St. Mark's Hospital in New York City; Gynecologist to the German Dispensary in the City of New York; Consulting Obstetrician to the New York Asylum (resigned); ex-President of the German Medical Society of the City of New York, etc., etc. 690 pages. Three hundred and ten engravings and colored plates. Price, cloth, $4; sheep, $5. W. B. Saunders, publisher, 925 Walnut street, Philadelphia, 1894.

Among the many recent works on diseases of women we have found none better suited to the medical student or the general practitioner than this one. It contains many practical points that are entirely wanting in works of greater magnitude, and it is these small matters that worry the young practitioner most. The purpose of the author in preparing this work was to supply a real need to the following classes of students and medical practitioners: First, to those "who have not had the advantage of hospital training, and *who go to a post-graduate school* in order to learn gynecology. They can only stay a short time, and they want a full and concise exposition, up to date, of the nature and treatment of the diseases peculiar to women.''

Secondly, he has tried to satisfy the wants of the much larger class of medical men who would like to attend a post-graduate school, but from various reasons find it impossible. These are men who are anxious to keep abreast of recent medical progress, but can not find time to study larger works.

The other class for which the book is especially designed is the under-graduate studying in medical colleges. The book has been written in such a way as to render it especially serviceable to all these classes, very little reference being made to pathology, and the reader's time is not taken up with theoretical discussions, but the author has endeavored to write a *practical* work, one which will help the physician to make a correct diagnosis and teach him how to treat the different diseases in accordance with the views of the best American authorities. The book is a most excellent one, and we heartily commend it to all general practitioners, and especially to those who have searched through the various works at their command for information on some so-called minor points and details in gynecological practice and have met with disappointment. To all such this volume will prove a boon indeed. H.

---

A SYSTEM OF LEGAL MEDICINE.—A Complete Work of Reference for Medical and Legal Practitioners, by Allan McLane Hamilton, M. D., Consulting Physician to the Insane Asylum, of New York City, etc., etc., assisted by Lawrence Godkin, Esq., of the New York Bar, with the collaboration of Prof. James F. Babcock, Lewis Balch, M. D., Judge S. E. Baldwin, Louis E. Binsse, Esq., C. F. Bishop, Esq., A. T. Bristow, M. D., B. F. Cardoza, Esq., C. G. Chaddock, M. D., A. F. Currier, M. D., C. L. Dana, M. D., W. S. Haines, M. D., F. A. Harris, M. D., George Ryerson Fowler, M. D., W. T. Gibb, M. D., W. B. Hornblower, Esq., Chas. Jewett, M. D., P. C. Knapp, M. D., R. C. McMurtrie, Esq., C. K. Wells, M. D., J. E. Parsons, Esq., C. E. Pellew, Ph. D., Judge C. E. Pratt, W. A. Purrington, Esq., B. Sachs, M. D., F. R. Sturgis, M. D., Brandreth Symonds, M. D., V. C. Vaughan, M. D. In two large royal octavo volumes of about 700 pages each. Illustrated. Price, in substantial cloth binding, per volume, $5.50; in full sheep, uniform law style, per volume, $6.50. Sold by subscription. Orders taken only for the complete work. E. B. Treat, publisher, 5 Cooper Union, New York City. Vol 1.

In an advance notice of this great work, published in the TEXAS MEDICAL JOURNAL, we stated to our readers that they might confidently expect one of the best books ever published on medial jurisprudence, and since examining this volume, we feel that the editor, collaborators and publisher have surpassed our anticipations. The following subjects are treated of in Vol. 1: Definition and History of Medical Jurisprudence, by Lawrence Godkin, Esq.; Medico-Legal Inspections and Post-Mortem Examinations, by Algernon T. Bristow, A. B., M. D.; Death in its Medico-Legal Aspect, by Francis A. Harris, M. D.; Blood and other Stains—Hair, by Prof. James F. Babcock; Identity of the Living, by Allan McLane Hamilton, M. D.; Identity and Survivorship, by Benjamin F. Cardoza, Esq.; Homicide and Wounds, by Lewis Balch, M. D., Ph. D.; Poisoning by Inorganic Substances, by Charles E. Pellew, Ph. D.; Poisoning by Alkaloids and Organic Substances, by Walter S. Haines, A. M., M. D.; the Toxicologic Importance of Ptomaines and other Putrefactive Products, by Victor C. Vaughan, Ph. D., M. D.; The Medical Jurisprudence of Life Insurance, by Brandreth Symonds, A. M., M. D.; Accident Insurance, by Cortlandt Field Bishop, Esq.; The Obligation of the Insured and the Insurer, by R. C. McMurtrie, Esq.; Of Certain Legal Relations of Physicians and Surgeons to Their Patients and One Another, by William Purrington, Esq.; Indecent Assault Upon Children, by W. Travis Gibb, M. D. The book is thoroughly American, and differs from the works on the same subject that have preceded it, in that it is the result of the combined labors of a number of America's greatest physicians and lawyers. Most of the articles written by the legal profession have been prepared with a member of the medical profession as collaborator. As a book of reference it will be found an invaluable help to physicians and lawyers, and especially to those who desire to become acquainted with the most advanced views of practical students of forensic medicine. It can justly be classed as one of the great books of this age.

